# Wound Healing Activities of Hydromethanolic Crude Extract and Solvent Fractions of *Bersama abyssinica* Leaves in Mice

**DOI:** 10.1155/2021/9991146

**Published:** 2021-07-14

**Authors:** Solome Melkamu Taddese, Tiruzer Bekele Gurji, Mohammedbrhan Abdulwuhab, Tezera Jemere Aragaw

**Affiliations:** ^1^Debre Tabor Health Science College, Debre Tabor, Ethiopia; ^2^Department of Pathology, College of Medicine and Health Sciences, University of Gondar, Gondar, Ethiopia; ^3^Department of Pharmacology, College of Medicine and Health Sciences, University of Gondar, Gondar, Ethiopia

## Abstract

**Background:**

*Bersama abyssinica* leaves are traditionally used for management of wounds in several communities of Ethiopia, despite no scientifically approved studies done on wound healing. Our study planned to work out the wound healing effects of *B. abyssinica* leaves extract in mice.

**Methods:**

*B. abyssinica* leaves were extracted with 80% hydromethanol and fractioned with chloroform, hexane, and water. The acute dermal toxicity of the crude extract was evaluated in mice. The crude extract formulated at 5% and 10% w/w ointment was investigated in excision, incision, and burn wound models and solvent fractions in the excision model using simple ointment (negative control) and nitrofurazone 0.2% w/v (positive control). We evaluated histopathological analysis, wound contraction rate, complete epithelialization period, and skin durability. One-way ANOVA followed by the post hoc Tukey HSD test with IBM SPSS software version 23.0 was used for data analysis, and *p* < 0.05 was considered statistically significant.

**Results:**

Hydromethanolic crude extract produced 5% (99.5%) and 10% (100%) wound contraction on the 16^th^ day of the treatment and 5% (18.8) and 10% (28.2) percent reduction in the epithelization period on the excision wound healing model. Hydromethanolic crude extract produced 5% (47.5) and 10% (61.17) percent durability on the incision wound healing model. Hydromethanolic crude extract produced 5% (99.82%) and 10% (100%) wound contraction on the 20^th^ day of treatment and 5% (13%) and 10% (21.7%) reduction in the epithelization period on the burn wound healing model. The chloroform fraction produced 5% (90.17%) and 10% (91.01%), hexane fraction produced 5% (85.81%) and 10% (86.78%), and aqueous fraction produced 5% (99.17%) and 10% (99.38%) wound contraction on the 14^th^ day of the treatment and 5% (18.8) and 10% (28.2) percent reduction in the epithelization period on the excision wound healing model. Both hydromethanolic crude extract and solvent fractions at 5% and 10% (w/w) were significant (*p* < 0.001) compared with negative control.

**Conclusion:**

The results of this study showed that both 5% w/w and 10% w/w of 80% hydromethanolic crude extract and solvent fractions of *B. abyssinica* leaves have wound healing effects.

## 1. Introduction

Wound is any disruption of the skin associated with microbial, physical, and chemical agents [1]. It is associated with morbidity, disability, socioeconomic crisis, and mortality [2]. Wound management is maintaining a warm, moist, nontoxic environment that supports natural wound healing [3]. Traditional medicine is an accepted, effective, easily available, and affordable treatment by applying knowledge, which incorporate mineral, animal, and plant based medicine to treat, diagnose, and maintain wellbeing [4–7]. Traditionally used medicinal plants have wound healing activity due to phytoconstituents with mechanism of analgesic proliferative, antioxidant, anti-inflammatory, and antimicrobial effects, which are crucial for management of wounds [8].

*B. abyssinica* Fresen is a shrub approximately 6 meters in height, which is evergreen and common in some African countries including Ethiopia [9]. Fruits powder of *B. abyssinica* mixed with butter has been used to treat eczema [10]. Leaves and roots are used for management of hypertension and cough [11]. Leaves decoction as tea accustomed for *Ascaris* and diarrhea [12–15]. Fresh Shoot tip chewed and swallowed for management of Stomachache and squeezed juice applied on the wound [16]. Stem bark taken orally to treat tonsillitis [17]. Warm leaves hold on affected area for rheumatics [18]. Buds were taken orally to manage snake bite [19]. Leaves, stems, and roots decoction were used for malaria and taeniafuge, stem barks were applied topically for snake bite, and fresh roots were boiled in water and consumed orally for febrile illness, bronchitis, and cancer [20, 21]. Roots and leaves decoction drunk with milk for rabies [13]. In Ethiopia, leafy-stem tips were squeezed and creamed on wound [22]. Leaves, stems, and roots decoction were applied on wound [23]. Leaves, roots, barks, and stems decoction were applied on wound [24]. Dry leaves were crushed or burned and mixed with butter for skin infection [25, 26]. Seed powders were applied on wound and skin burn [27].

In vitro hydromethanolic and ester leaf extract of *B. abyssinica* exhibited higher activity against *Klebsiella pneumonia, Staphylococcus aureus, Escherichia coli,* and *Salmonella typhosa* [9]. Aqueous extract of stem bark of this plant had bactericidal activity against multiresistant strains of *Staphylococcus aureus* [28]. Leaf, stem bark, and root bark hydromethanolic extracts of *B. abyssinica* had higher antibacterial activities against two fast growing bacteria like *M. madagascariense* and *M. indicus pranii* at 0.039 and 0.78 mg/mL, respectively [29]. Hydromethanolic stem bark crude extract of *B. abyssinica* possesses much higher antibacterial activity against *Escherichia coli, Staphylococcus aureus, Klebsiella pneumonia*, *Bacillus subtilis*, and *Pseudomonas aeruginosa* [30].

Hydromethanolic leaves extract of this plant had significant activity in scavenging of free radical, and 50 percent inhibitory concentration is 7.5 *µ*g/ml [31]. Aqueous and hydroethanolic leaf extract possesses an antifungal activity with maximum effect of hydroethanolic extracts (minimum inhibitory concentration = 0.781 mgml^−1^ and 50 percent inhibitory concentration is 0.08 mgml^−1^) [32].

Phytochemical screening of *B. abyssinica* leaves extract revealed the presence of coumarins, terpenoids, saponins, polysterols, anthraquinones, triterpenes, tannins, phenols, steroids, flavonoids, glycosides, and alkaloids, whereas anthraquinones, saponins, and terpenoids were highly concentrated in methanolic extract [9, 33–37].

## 2. Rationale of the Study

*B. abyssinica* is utilized as wound healing agent in several communities of Ethiopia. It has been scientifically investigated for wound healing efficacy in vitro, but not in vivo. In vivo evaluation of this plant is also important to suggest safety and efficacy for continuous use in the community and can be a starting point for scientific communities to undergo further investigation for the development of effective as well as safe drug used for wound healing.

## 3. Materials and Methods

### 3.1. Drugs and Chemicals

Drugs and chemicals like Wool fat (Lab tech chemicals), hard paraffin (Lab tech chemicals), white petrolatum (Lab tech chemicals), cetostearyl alcohol (Lab tech chemicals), methanol absolute (Sisco Research lab pvt. ltd), chloroform (Carlo Erba reagents), hexane (Pentokey Organy India ltd), distilled water, nitrofurazone skin ointment 0.2% (Shanghai general pharmaceutical), ketamine hydrochloride injection USP(India), diazepam injection (Gland pharm limited, India), 70% alcohol (Yilmana chemical production), normal saline (IV infusion BP Medsol pharmaceuticals), 10% formalin solution were procured from supplier and used.

### 3.2. Plant Material Collection and Preparation

*Bersama abyssinica* leaves were collected near Debre Tabor town (Alem Saga), at a latitude and longitude of 11°51′N, 38°1′E and 2706 meters above sea level on January 11, 2020. Specimen of *B. abyssinica* was identified taxonomically by a Botanist at the Department of Biology, College of Natural and Computational Science, University of Gondar, Gondar, Ethiopia, and registered with a reference number of 001SMT/2020.

### 3.3. Experimental Animals

A total of 126 healthy adult swiss albino mice of weight 25–35 g and age 6–8 weeks of either sex for the actual experiment and 10 healthy female adult swiss albino mice for determining acute dermal toxicity test that were inbred at the Animal House of Department of Pharmacology, School of Pharmacy, College of medicine and Health Sciences, University of Gondar, Gondar, Ethiopia, were used. Mice were kept (five mice/cage) in clean polyethylene plastic cages with a wire mesh top containing a hygienic bed of course sawdust (regularly changed every 3 days) and retained in a well-ventilated room 24–25°C, 50–60% humidity and a light-dark cycle of 12 hours with free delivery of standard pellet diet purchased from local suppliers and clean potable tape water. Then, the mice were acclimatized with the test environment for a week before the initiation of the experiment. The care and handling of mice were performed with globally accepted standard laboratory protocols and guidelines [38, 39]. And research was performed as per the agreement of the ethical clearance document Ref. No SoP4/153/12.

### 3.4. Preparation of the Hydromethanolic Crude Extract

To obviate contaminants and debris leaves, they were washed with water and shade-dried at room temperature and coarsely powdered with a hand compression to maximize penetration of extracting solvents. Powder material of plant was packed in tightly closed container until extraction. A coarsely powdered 1000 gm leaves of *Bersama abyssinica* were macerated in 6 liters of 80% hydromethanolic solution in Erlenmeyer flasks for 3 days with frequent shaking in between. After 3 days of maceration, extraction was performed using thick layers of 40 mesh gauze and filtered with Whatman paper No. 1. The residue was remacerated for 3 days two times using similar volume of 80% hydromethanolic solution to induce maximum yield. The filtrate was frozen overnight using deep-freeze and freeze-dried in a lyophilizer at −50°C to eliminate water. The proportion yield of extract was determined and stored in a refrigerator at 4°C up to ointment formulation and solvent fractionation [40].

(1) $$\begin{array}{c} \text{Percent yield }=\;\frac{\text{weight of the extract}}{\text{weight of the plant material}}\times 100. \end{array}$$

### 3.5. Solvent Fractionation of Hydromethanolic Crude Extract

Hydromethanolic crude extract of *Bersama abyssinica* leaves was processed for further fractionation using water, chloroform, and hexane. 420 ml of water was added to 70 gram hydromethanol in a separatory funnel. Then, equal volume of hexane was added thereto. After being gently shaken, the extract was allowed to settle and separated into two distinct layers according to their density. The upper hexane layer was collected, and thus identical procedure was repeated thrice. After the gathering of hexane fraction of the extract, the water portion was fractionated with chloroform 3 times with the same procedure of hexane, and so, the underside chloroform layer was collected, and finally, aqueous fraction was collected. The filtrates of chloroform and hexane fractions were concentrated by rotary evaporator and dried by dry oven at 40°C. The aqueous fraction was frozen in refrigerator overnight and was dried with lyophilizer. The proportion yield was calculated and stored at 4°C until ointment formulation [40].

### 3.6. Ointment Formulation

Simple ointment and medicated ointments of 80% hydromethanolic crude extract, chloroform, hexane, and aqueous fractions were prepared per British Pharmacopeia [41].

Simple ointment base 300 gm was prepared by placing 15 gm of hard paraffin in a beaker and melted on electrical heater. Then, 15 gm of Cetostearyl alcohol, 255 gm of white petrolatum, and 15 gm of wool fat were added in descending order of their temperature, respectively. All the ingredients were melted over electrical heater with constant stirring until they became homogeneous. Then, the mixture was removed from heat source and stirred until being cool. All ointments were prepared by levigation on the surface of the ointment slab to formulate ointment of uniform consistency and smooth texture. For negative control, 100 gm of simple ointment base was prepared without active ingredient. Finally, hydromethanolic crude extract and solvent fractions ointment and simple ointment base were transferred to a clean closed container for topical application during the experiment [42] (see Table 1).

### 3.7. Acute Toxicity Study

Acute oral toxicity was previously done in mice. The findings of this study showed that a limited dose of 2000 mg/kg was safe, and a median lethal dose (LD_50_) of the extract was greater than 2000 mg/kg [40, 43]. Acute dermal toxicity test of 80% hydromethanolic crude extract of *Bersama abyssinica* was performed in accordance with OECD guideline 425 with slight modification [44]. Ten healthy Swiss albino female mice showing normal skin texture were used. Mice were grouped into two, with five mice for control and five mice for test group. 24 hours before the study, 10% of surface fur of test mouse was removed with scissors from the dorsal area of the trunk. Initially sighting study was performed on one mouse to evaluate the starting dose by applying 2000 mg/kg of the ten percent formulation of the hydromethanolic crude extract uniformly over the shaved area and covered with surgical gauze and a nonocclusive bandage for 24 hours. During the exposure period, mouse was caged individually. At the end of the exposure period, residual test substance was washed out by water. Irritation or death was not observed within 24 hours. Then, the remaining four mice from each group were tested on the following day with limited test dose of 2000 mg/kg of 10% ointment formulation of the extract and observed for 24 hours. At the moment, mice were observed for development of any adverse skin reactions immediately after dosing, for half an hour, periodically during the first 24 hours, with special attention given during the first four hours, and daily for a total of 14 days [45].

### 3.8. Grouping and Dosing of Experimental Animals

Hydromethanolic crude extract was evaluated with excision, incision, and burn wound healing models, while solvent fractions were evaluated with excision wound healing model only. In excision and burn wound models, four groups of mice containing six mice in each group were used. Group-I was treated with hydromethanolic crude extract 5% w/w ointment, group-II treated with hydromethanolic crude extract 10% w/w ointment, group-III treated with nitrofurazone 0.2% w/v ointment (positive control), and group-IV treated with simple ointment (negative control). The identical grouping and dosing of mice were also used for incision wound healing model except addition of fifth group for untreated group [46].

For evaluation of solvent fractions (aqueous, chloroform, and hexane) in excision wound healing model, eight groups of mice containing six mice in each group were used. Group-I was treated with aqueous fraction 5% w/w ointment, group-II treated with aqueous fraction 10% w/w aqueous ointment, group-III treated with chloroform fraction 5% w/w ointment, group-IV treated with chloroform fraction 10% w/w chloroform fraction ointment, group-V treated with hexane fraction 5% w/w ointment, group-VI treated with hexane fraction 10% w/w ointment, group-VII treated with nitrofurazone 0.2% w/v ointment (positive control), and group-VIII treated with simple ointment (negative control) [47].

### 3.9. Circular Excision Wound Model

**Figure 1:**
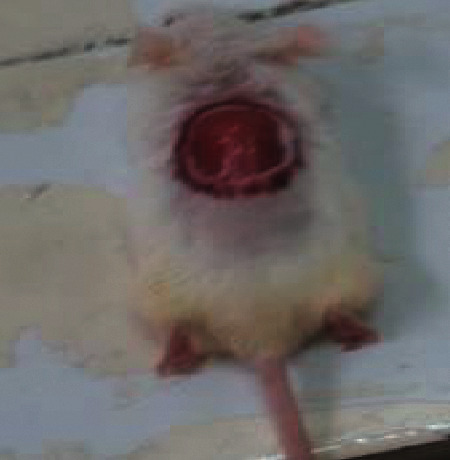
Photograph of circular excision wound on day 0.

For wounding purpose, the mice were anesthetized with intraperitoneal (IP) injection of ketamine + diazepam (100/5 mg/kg) [48]. Wound area was cleaned with 70% denatured hydroethanol, and also the dorsal furs of the mice were removed with a pair of scissors. Then, the estimated wound area was marked with thin permanent marker, and a full thickness excision wound around 300 mm^2^ was excised using forceps and small sharp sterilized pair of scissors to each mouse (Figure 1). Bleeding of wound was stopped by blotting the wound with cotton swab soaked with normal saline and left open. After recovery, each mouse was returned back to its cage and thought to be day-0 wounding day. Starting from day one, the wounded area was treated daily as described under dosing section until the wound of test groups completely healed. The wound healing activity of hydromethanolic crude extract and solvent fractions was assessed by period of epithelialization, percentage of wound contraction, and histological studies [45].

### 3.10. Wound Healing Parameters

#### 3.10.1. Wound Contraction

Wound contraction was evaluated by measuring wound areas using transparency paper and permanent marker. The measured area was important to calculate percentage of wound contraction and taken initial size of the wound, 300 mm^2^ as 100% with the following formula [49]:

(2) $$\begin{array}{c} \text{\% wound contraction}=\frac{\text{initial wound size }-\text{wound size on day }n}{\text{initial wound size }}\times 100, \end{array}$$

where *n* = the days where measurement was taken (2^nd^, 4^th^, and so forth).

#### 3.10.2. Epithelization Period

Epithelization period was resolute by the number of days required for declension of the dead tissue remnants with none residual lesion [50].

### 3.11. Histopathological Analysis

Histopathological analysis was performed blindly by a pathologist. The skin specimens from each group were collected at the 18^th^ postwounding day of the experiment after the mice are sacrificed by overdose of anesthesia. Samples were fixed in 10% buffered formalin, processed, and blocked with paraffin and stained with hematoxylin and eosin. The tissues were examined by microscope and graded subjectively as mild (+), moderate (++), and severe (+++) for epidermal or dermal remodeling, reepithelialization; fibroblast proliferation, mononuclear, and/or polymorphonuclear cells and collagen depositions in dermis were analyzed [49, 50].

### 3.12. Linear Incision Wound Model

**Figure 2:**
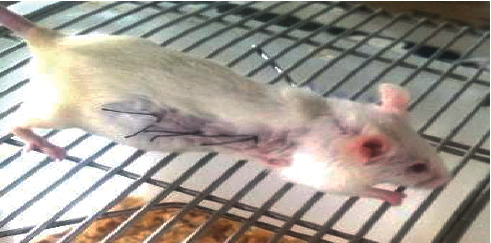
Photograph of incision wound on day 0.

The mice were anesthetized and shaved like circular excision wounds. Then, 3 cm long longitudinal paravertebral incision was made through the skin and subcutaneous tissue. The parted skin was stitched by nonabsorbable surgical thread and a curved needle at the intervals of 1 cm (Figure 2). After 24 hours of wound creation (on day-1), mice were treated as described under dosing section. The sutures were removed on day-9, and strength of the healed wound was measured on day-10 by continuous and constant water flow technique [45, 49].

### 3.13. Measurement of Tensile Strength

**Figure 3:**
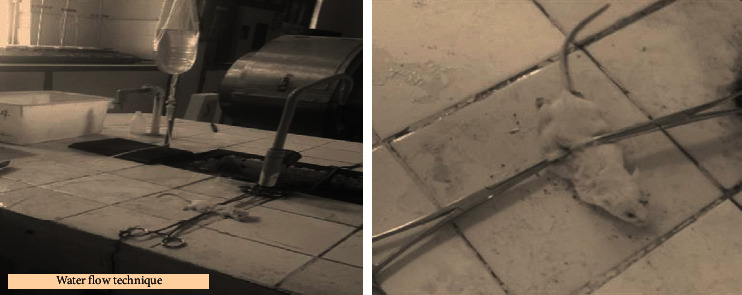
Photograph of the measurement of tensile strength by using water flow technique.

Mice were anesthetized and secured to the table. Two forceps were firmly applied 3 mm away from the edge of wound facing one another on opposite side of the incision wound. One among the forceps was fixed on stands, while the opposite was tied with rope to empty bottle, on which weight was placed. Water was allowed to flow continuously and slowly from IV bag filled with 1000 ml H_2_O into the container (Figure 3). The water flow was stopped immediately, just the wound unfolded and therefore, the volume of water collected in the container (approximately equal to its weight) was recorded as tensile strength [45, 49].

Percent of durability was calculated using the subsequent formula:

(3) $$\begin{array}{c} \text{ percent tensile strength }\left(\text{TS}\right)\text{ of the extract}=\frac{\text{TS extract}-\text{TS so}}{\text{TS so}}\times 100,\\ \text{percent tensile strength of the reference}=\frac{\text{TS reference}-\text{TS so}}{\text{TS so}}\times 100,\\ \text{percent tensile strength of simple ointment}=\frac{\text{TS So}-\text{TS lu}}{\text{TS lu}}\times 100, \end{array}$$

where so = simple ointment and Lu = left untreated [50].

### 3.14. Burn Wound Model

**Figure 4:**
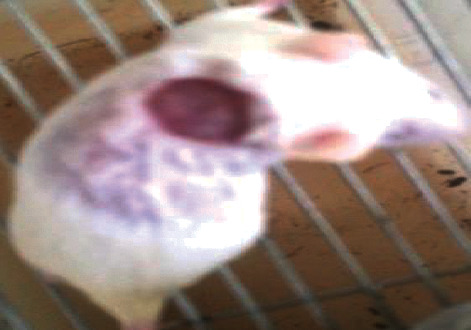
Circular burn wound on day 0.

Mice were divided randomly into four groups of six mice in each group, then anesthetized, and shaved like excision wound model. Following this, partial thickness burn wound was created by pouring hot molten waxes at 80°C into a metal cylinder of 300 mm^2^ circular opening placed on the shaved area of the mice and stayed until wax got solidified for 10–12 minutes. On solidification of wax, the metal cylinder with wax that adheres to the skin was removed (Figure 4). After recovery, mice were returned to their cage and treated as described under dosing section. Wound healing parameters like period of epithelialization, percentage of wound contraction, and histological studies were assessed [51].

### 3.15. Statistical Analysis

The results obtained from the experiments were expressed as mean ± SEM. The result was statistically analyzed using one-way ANOVA followed by post hoc Tukey tests with SPSS version 23.0 software, and *p* < 0.05 was considered as statistically significant.

## 4. Result

### 4.1. Yield of Crude Extract and Solvent Fractions

High yield was obtained from aqueous fraction (64.3%), and lower yield was obtained from n-hexane fraction (3.6%) (see Table 2).

### 4.2. Acute Dermal Toxicity

**Figure 5:**
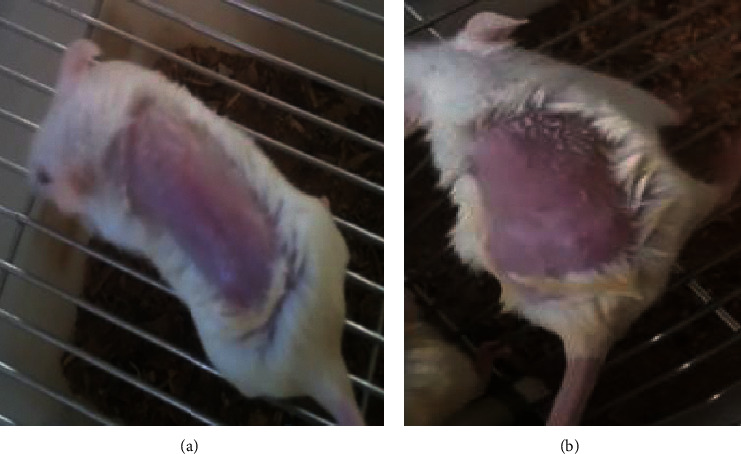
Photographs of acute dermal toxicity test results: (a) hydromethanolic crude extract 10% w/w and (b) simple ointment.

The acute dermal toxicity study indicated that topical administration of maximum concentration of the hydromethanolic crude extract of *Bersama abyssinica* leaves ointment formulation (10% (w/w)) with a limit dose of 2000 mg/kg on animals did not show any sign of dermal toxicity as depicted in Figure 5(a) and simple ointment observed in Figure 5(b). With 6-hour special attention and 14-day cage side observation periods, animals showed no sign of edema, erythema, and death (see Figure 5).

## 5. Wound Healing Activity of Crude Extracts

### 5.1. Excision Wound Model

#### 5.1.1. Wound Contraction

Hydromethanolic crude extract ointment formulations of *B. abyssinica* leaf increased wound contraction in excision wound model compared with mice treated with simple ointment base. Hydromethanolic crude extract of *Bersama abyssinica* leaves 10% w/w and nitrofurazone 0.2% ointment enhanced wound contraction significantly (*p* < 0.01) at 4^th^ postwounding day and more significant (*p* < 0.001) from 6^th^ postwounding day onwards as compared with negative control. Likewise, 10% w/w hydromethanolic crude extract of *Bersama abyssinica* leaves showed significant (*p* < 0.001, *p* < 0.01) wound contraction from 8^th^ to 12^th^ and 14^th^ postwounding days, respectively, compared with mice treated with 5% w/w crude extract ointment. On the other hand, the mice treated with 5% (w/w) extract ointment showed significant wound contraction from 4^th^ to 6^th^ (*p* < 0.05), 8^th^ (*p* < 0.01), and 10^th^ postwounding day onwards (*p* < 0.001) as compared with mice treated with simple ointment. Slightly higher rate of wound contraction was observed with 10% w/w crude extract ointment than reference nitrofurazone, but not statically significant (see Table 3).

Higher and comparable percentage of wound contraction on excision wound healing model was observed in groups treated with 5%, 10% w/w hydromethanolic crude extract of *Bersama abyssinica* leaves and nitrofurazone 0.2% w/v at 12^th^ (90.58%, 93.73%, and 94.18%), 14^th^ (99.29%, 99.35%, and 97.05%), and 16^th^ (99.55%, 100%, and 100%) postwounding days, respectively, and was highly significant (*p* < 0.001) compared with negative control. Complete wound closure was observed in hydromethanolic crude extract 10% of *Bersama abyssinica* leaves and nitrofurazone 0.2% w/v at 16^th^ postwounding days, respectively (see Table 4).

#### 5.1.2. Epithelization Period

Topical application of both crude extract ointment and reference drug showed significant reduction in epithelialization period (*p* < 0.001) as compared with mice treated with simple ointment. Time for complete epithelization was reduced by 28.2% for 10% (w/w), 18.8% for 5% (w/w), and 27.4% for 0.2% nitrofurazone ointments, respectively. This is evidenced by shorter period for fall of scab (see Table 5).

#### 5.1.3. Histopathological Analysis

**Figure 6:**
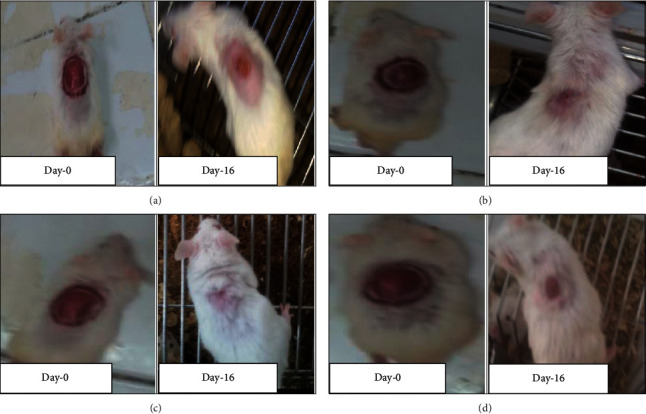
Photographic comparison of wound contraction on mice treated with simple ointment, nitrofurazone ointment, and hydromethanolic crude extract on excision wound model. (a) Mice treated with simple ointment base. (b) Mice treated with hydromethanolic extract 5% w/w ointment. (c) Mice treated with hydromethanolic extract 10% w/w ointment. (d) Mice treated with nitrofurazone 0.2% w/v ointment.

**Figure 7:**
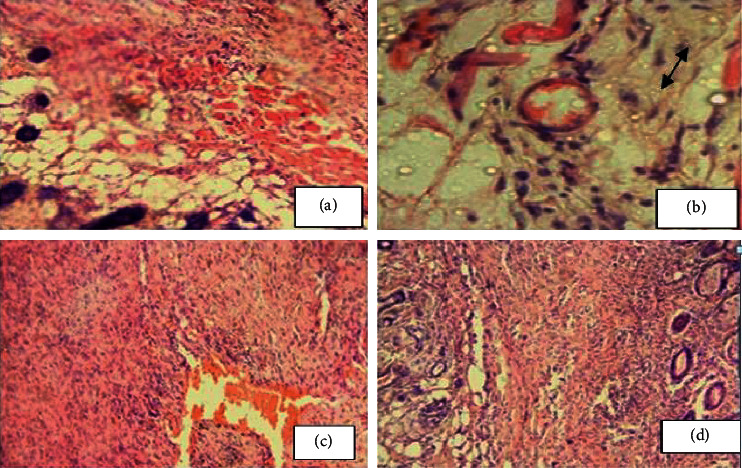
Photograph of hematoxylin and eosin staining of tissues from partial thickness burn wound model in mice. (a) Mice treated with simple ointment. (b) Mice treated with 5% crude extract ointment. (c) Mice treated with 10% crude extract ointment. (d) Mice treated with nitrofurazone.

Granulation tissue taken from 10% w/w hydromethanolic crude extract treated group showed no inflammatory cells, more collagen fiber, more fibroblasts proliferation, and less blood capillaries (angiogenesis). 5% w/w hydromethanolic crude extract treated group showed few inflammatory cells, moderate collagen fiber, fibroblasts proliferation, and less blood capillaries (angiogenesis). The reference drug treated group also showed no inflammatory cells, less collagen fiber, less fibroblasts proliferation, and less blood capillaries (angiogenesis) as compared with mice treated with simple ointment. Simple ointment treated group showed less collagen fibers, less fibroblasts proliferation, and no blood capillaries, and more inflammatory cells, thus showing delayed wound healing processes (see Table 6). And photographic comparison of the wound on day 0 (wound creation) and day 16 after treatment of simple ointment, hydromethanolic crude extract, and nitrofurazone ointment is shown in Figure 6 and Photograph of hematoxylin and eosin staining of tissues from partial thickness burn wound model in mice is shown in Figure 7.

### 5.2. Linear Incision Wound Healing Study

#### 5.2.1. Tensile Strength

Both 5% w/w and 10% w/w hydromethanolic crude extract and nitrofurazone ointment treated group showed significant (*p* < 0.001) increase in tensile strength as compared with simple ointment base and untreated groups. Percent tensile strength of 10%, 5% (w/w) hydromethanolic crude extract ointments and nitrofurazone 0.2% ointment treated group was 61.17, 47.5, and 62.62, respectively (see Table 7).

### 5.3. Burn Wound Healing Activity

#### 5.3.1. Wound Contraction

Hydromethanolic crude extract at 5% w/w, 10% w/w, and nitrofurazone 0.2% ointment showed enhanced wound contraction in partial thickness burn wound model as compared with mice treated with simple ointment. Hydromethanolic crude extract at 10% w/w and nitrofurazone 0.2% ointment started to show significant (*p* < 0.01) wound contraction at 4^th^ postwounding day and enhance it more significantly (*p* < 0.001) from 6^th^ postwounding day onwards as compared with simple ointment base. Likewise, hydromethanolic crude extract 10% w/w showed significant (*p* < 0.05 to 0.01) wound contraction from 6^th^ to 18^th^ postwounding days as compared with hydromethanolic crude extract 5% w/w. On the other hand, hydromethanolic crude extract 5% w/w recorded significant wound contraction from 6^th^ (*p*< 0.05) and 8^th^ postwounding day onwards (*p* < 0.001) as compared with simple ointment treated mice. Reference drug showed slightly higher rate of wound contraction as compared with hydromethanolic crude extract 10% w/w but lacks statistical significance. Complete wound closure was observed in hydromethanolic crude extract and nitrofurazone 0.2% ointment at 20^th^, 20^th^, and 20^th^ postwounding days, respectively, but simple ointment treated animal was beyond 22^nd^ postwounding day (see Table 8).

Higher and comparable percentage of wound contraction was recorded in groups treated with 5%, 10% w/w hydromethanolic crude extract, and nitrofurazone at 12^th^ (63.6%, 68.17%, and 69.27%), 14^th^ (82.34%, 81.47%, and 77.08%), and 16^th^ (91.97%, 90.42%, and 87.71%) postwounding days respectively. Complete wound closure was observed in nitrofurazone 0.2%, 10% (w/w), 5% (w/w) hydromethanolic crude extract and simple ointment treated groups were at 20^th^, 20^th,^ 22^nd^, and 24^th^ postwounding days, respectively (see Table 9).

### 5.4. Epithelization Period

**Figure 8:**
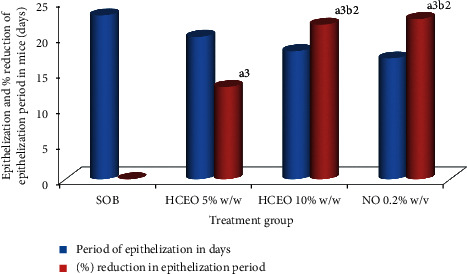
Effect of hydromethanolic crude extract of *B. abyssinica* on period of epithelialization and percent reduction in epithelization period on partial thickness wound model in mice. SOB = simple ointment base, HCEO = hydromethanolic crude extract ointment, NO= nitrofurazone ointment. (a) Compared with simple ointment; (b) compared with 5% w/w: 2*p* < 0.01, 3*p* < 0.001.

Hydromethanolic crude extract 10% w/w crude extract ointment treated animals showed significant (*p* < 0.01, *p* < 0.001) reduction of epithelization period as compared with hydromethanolic crude extract 5% and simple ointment treated mice, respectively. Likewise, hydromethanolic crude extract 5% w/w decreases epithelization period significantly (*p* < 0.001) to mice treated with simple ointment. Hydromethanolic crude extract 10%, 5%, and nitrofurazone 0.2% ointment reduce epithelization period with 21.7%, 13%, and 22.5%, respectively. However, epithelization periods of hydromethanolic crude extract 10%w/w extract ointment and nitrofurazone 0.2% ointment were not statistically significant (see Figure 8).

### 5.5. Histopathological Analysis

**Figure 9:**
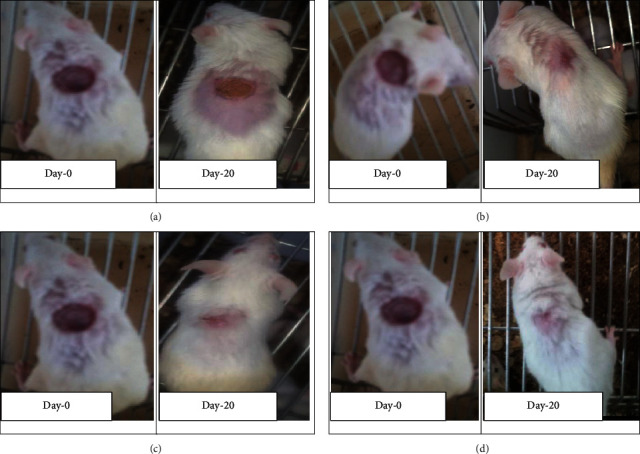
Photographic comparison of wound contraction on mice treated with simple ointment, nitrofurazone ointment, and hydromethanolic crude extract on burn wound model. (a) Mice treated with simple ointment base. (b) Mice treated with hydromethanolic extract 5% w/w ointment. (c) Mice treated with hydromethanolic extract 10% w/w ointment. (d) Mice treated with nitrofurazone 0.2% w/v ointment.

Granulation tissue taken from 10% w/w hydromethanolic crude extract treated group showed few inflammatory cells, modest collagen fiber, less fibroblasts proliferation, and modest blood capillaries (angiogenesis). 5% w/w hydromethanolic crude extract treated group showed few inflammatory cells, few collagen fiber, and fibroblasts proliferation and less blood capillaries (angiogenesis). The reference drug treated group also showed less inflammatory cells, modest collagen fiber, modest fibroblasts proliferation, and moderate blood capillaries (angiogenesis) as compared with simple ointment treated group. Simple ointment treated group showed less collagen fibers, less fibroblasts proliferation, and more inflammatory cells, thus showed delayed wound healing processes (see Table 10). And photographic comparison of the wound on day 0 (wound creation) and day-16 after treatment of simple ointment, hydromethanolic crude extract, and nitrofurazone ointment is shown in Figure 9.

## 6. Wound Healing Activity of Solvent Fractions

### 6.1. Wound Contraction

All of the solvent fractions ointment preparations promoted wound healing as compared with mice treated with simple ointment. From 4^th^ postwounding day onwards, aqueous fraction ointments (10% w/w and 5% w/w) and nitrofurazone 0.2% ointment recorded significant (*p* < 0.001) wound contraction as compared with simple ointment. Both chloroform fraction ointments showed significant (*p* < 0.05 to 0.001) wound contraction from 4^th^ to 16^th^ postwounding days as compared with negative control. Likewise, 10% w/w chloroform fraction ointment recorded significant (*p* < 0.05 to 0.001) wound contraction as compared with both hexane (5% w/w and 10% w/w) fraction ointment treated groups. From 12^th^ to 16^th^ postwounding days, 5% w/w and 10% w/w hexane fraction ointment enhanced wound contraction more significantly (*p* < 0.001) as compared with simple ointment. There was a slightly higher effect with (10% w/w) ointment preparations than (5% w/w) ointment preparations between each solvent fractions, but lacking statistical significance between high and low strengths. Similarly, there was modest difference between both aqueous fraction and reference drug, but not significant (see Table 11).

Higher percentage of wound contraction was observed from 14^th^ postwounding day on wards on mice treated with aqueous fraction (10% w/w, 5% w/w) and nitrofurazone 0.2% ointment, and the remaining fraction ointments were shown at 16^th^ postwounding day. Complete wound closure of aqueous fraction (5 and 10%), chloroform fraction (5 and 10%), hexane fraction (5 and 10%), and nitrofurazone 0.2% was 16^th^, 17^th^, 18^th^, and 16^th^ postwounding days, respectively, but negative control wound closure was beyond 18^th^ postwounding day (see Table 12).

### 6.2. Epithelization Periods of Fraction

**Figure 10:**
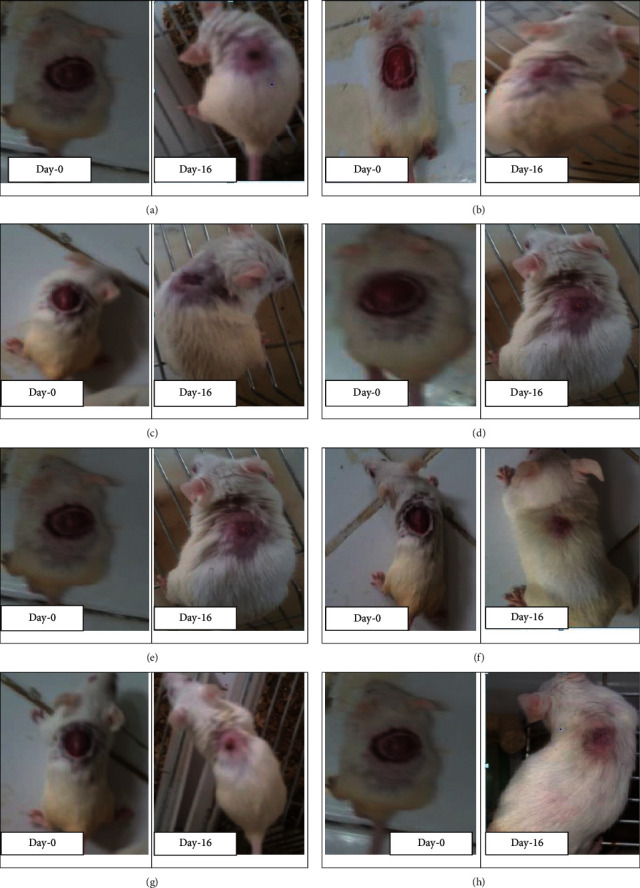
Photographs of excision wound model on day-0 and treated with solvent fractions of *B. abyssinica* leaves at 16th day post treatment (AF = aqueous fraction, CF = chloroform fraction, HF = hexane fraction). (a) Mice treated with simple ointment. (b) Mice treated with AF of *B. abyssinica* 5% w/w ointment. (c) Mice treated with AF of *B. abyssinica* 10% w/w ointment. (d) Mice treated with CF of *B. abyssinica* 5% w/w ointment. (e) Mice treated with CF of *B. abyssinica* 10% w/w ointment. (f) Mice treated with HF of *B. abyssinica* 5% w/w ointment. Mice treated with AF of *B. abyssinica* 10% w/w ointment. (h) Mice treated with nitrofurazone 0.2% w/v ointment.

All 5% w/w and 10% w/w fraction ointments could shorten the period of epithelialization against the animals treated with simple ointment. Animals treated with aqueous (5% and 10%) w/w fraction ointments and reference drug could shorten epithelization period more significantly (*p* < 0.001) as compared with simple ointment treated group and other fractions. Both strengths of hexane fraction ointment were significant (*p* < 0.05) as compared with negative control. There was no significant difference between the treatment doses of the three fractions, as well as with the positive control. Percentage reduction in epithelization period was higher for 5% water fraction (28.6%) and minimum for 5% hexane fraction (7.1%). There was a modest difference between epithelization period of 5% and 10% preparations of each fraction, but lacking significance when compared with each other (see Table 13). And photographic comparison of the wound on day 0 (wound creation) and day-16 after treatment of simple ointment, hydromethanolic crude extract, and nitrofurazone ointment was shown in Figure 10.

## 7. Discussion

Wound healing may be a fancy process, involving many biochemical process and cellular reactions. In wound management, sensitivity and skin irritations are common side effects, which diminish the speed of skin repair and increase the healing periods [52].

Traditionally, many medicinal plants support the speed of wound healing with minimal pain, discomfort, and scarring to the patient [47]. Majority of these plants are scientifically validated for their wound healing effects. The experimental plant *B. abyssinica* is among these plants traditionally used as a wound healing agent in several communities of Ethiopia. Except this study, no prior scientific evaluation in vivo was conducted for its wound healing activities. Therefore, this study was performed to scientifically assess the potential of this plant as an alternate treatment in wound healing in vivo. In vivo models are the foremost predictive models for studying wound healing, allowing a wise representation of the wound environment including various cell types and environmental cues. We chose mice for this study by considering cost and availability of them in conducting preliminary pharmacological screening [52].

For evaluation of plant wound healing effect in mice model, applying the fabric on to the wounded area cannot bring the desired effect. Hence, first, the leaves of *B. abyssinica* were extracted with 80% hydromethanol with maceration technique to separate medicinally active portion from insoluble cellular marc. Methanol was chosen for extraction because it is a solvent and has produced many phytochemicals of this plant from previous studies. Maceration technique is additionally more applicable, convenient, and among fewer costly methods for little and medium plant extractions [9, 53].

Topical application of plant extract only at wound site is additionally easily removed. It must be incorporated in simple ointment bases to achieve the specified effect by sustained release of extract at the application site. Ointment bases may form barrier for moisture over the wound area, which is crucial for cellular migration and diffusion of growth factors to wound [45].

No edema and irritation on mice observed with acute dermal toxicity test could indicate the security of the plant to be used as wound management. As explained above, irritation is the main side effect of wound managements.

Evaluation of *B. abyssinica* leaves hydromethanolic crude extract wound healing effect in an exceedingly single wound model was inadequate, and there is no reference standard, which could collectively represent the various components of wound healing phases [54]. Hence, different wound healing models, namely, linear incision, circular excision, and partial thickness burn wound models, were used.

In the present study, various parameters like wound contraction, epithelization, and strength were assessed in the above explained wound models to determine the speed of healing. The results of the study on excision wound model indicated that wound contraction rate observed from 10% (w/w) hydromethanolic crude extract ointment treated group was resembling the standard drug nitrofurazone, more significant than negative control, and better than 5% w/w hydromethanolic crude extract for several postwounding days. Likewise, 5% w/w hydromethanolic crude extract reduces wound area significantly from 8th postwounding day onwards as compared with negative control.

The finding of burn wound study showed that wound contraction rate observed in 10% (w/w) hydromethanolic crude extract ointment treated group was slightly different from that of nitrofurazone, more significant than negative control altogether postwounding days, and 5%w/w hydromethanolic crude extract ointment for several postwounding days.

Wound Contraction is also a movement of wound edges toward each other in a centripetal fashion. This is often important to decrease the wound's dimensions, reduce the amount of extracellular matrix needed to repair the defect, and desire less granulation to exchange tissue loss [55]. The enhanced wound contraction of hydromethanolic extract was probably because of inhibition of microbial growth. This was evidenced by previous in vitro studies of *B. abyssinica* hydromethanolic leaves extract that exhibited much higher antibacterial activity against wound infecting pathogenic bacteria, namely, *Staphylococcus aureus*, *Escherichia coli*, and *Klebsiella pneumoniae* [9].

Disruption of skin with burn and other injuries causes subsequent immune suppression, which favors the above explained bacteria. These bacteria and endotoxins may end up in increase proinflammatory cytokines and make the inflammatory phase longer. Prolonged inflammation also results in an increased level of matrix Metalloproteases (MMPs), which might degrade growth factors rapidly [56]. So, eradication of the colonizing organisms with hydromethanolic crude extract from wound area allows an appropriate environment for wound healing process. *B. abyssinica* leaves may have mitogenic activity, which reinforces fibroblast motility and cellular proliferation. This was evidenced by histopathological analysis result that showed the presence of fibroblast proliferation and collagen deposition in animal tissue of hydromethanolic crude extract ointment group treated mice. Previous in vitro studies of *B. abyssinica* leaves on Human foreskin fibroblast also showed mitogenic activity [28]. In step with these evidences, fibroblasts stimulate the assembly or synthesis of collagen. Collagen facilitates the migration of endothelial cells to create new blood vessels to strengthen granulation formation and consequently improve wound healing, which is observed as reduction in wound area. Similarly, fibroblasts rapidly show the activated phenotype of myofibroblast, which attributes higher cellular contractility that promotes the faster closure of the wound [43, 57].

The other reason for enhanced wound contraction effect is the antioxidant activity. This is often evidenced by in vitro antioxidant activity of hydromethanolic extract of *B. abyssinica* leaves [31, 43].

Mainly, burns manifest into systemic problems like hypermetabolism. The hypermetabolic cascade seems to involve: glucose metabolism with insulin resistance (IR) and hyperglycemia. High glucose is additionally increased due to production of free radicals and decreased levels of antioxidants. High glucose level affects wound healing by delaying proliferation of cells and reduce the assembly of collagen [58]. Hence, the use of antioxidants and antidiabetic may have a positive effect on the return of skin tissue damage and other burn wound complications. On the other hand, the typical period of epithelization for excision wound model was significantly reduced from negative control (19.5), 5%w/w hydromethanolic crude extract ointment (15.83), to 10% hydromethanolic crude extract ointment (14) days. Likewise, epithelization period of burn wound model was reduced significantly with 10%w/w hydromethanolic crude extract ointment from 23 days in negative control group to 18 days.

Epithelialization may be a process of covering defect on the epithelial surface during the proliferative innovate, in which keratinocytes undergo a series of migration, proliferation, and differentiation. The reduced period of epithelialization obtained by the hydromethanolic crude extract ointments is due to rapid wound contraction, as contraction shortens the space for migrating keratinocytes decreases [47, 59]. The opposite could even be due to the enhancement of collagen deposition by the hydromethanolic crude extract that facilitated proliferation, migration, and increased viability of epithelial cells [60], which is supported by histopathological analysis result.

Further wound healing activity of the hydromethanolic crude extract was evidenced by increasing durability in incision wounds. The lastingness of both 5%w/w and 10%w/w hydromethanolic crude extract ointment treated groups was highly significant (*p* < 0.001) as compared with simple ointment treated and untreated groups. Tensile strength indicates what proportion of the repaired tissue resists tensile under tension and can indicate the standard of repaired tissue and enhanced collagen maturation by increased cross-linking [49]. The mitogenic activity and fibroblast proliferation could even possibly increase enduringness [28]. Fibroblasts produce large quantities of collagen, which forms the foremost constituent of the extracellular wound matrix and which is ultimately accountable for making lastingness, which sticks the wound edges together at the repaired site [61].

The other could even be due to the presence of secondary metabolites like saponins and flavonoids that also are responsible for enhancement of collagen maturation, which provides strength and integrity to the wound matrix [9, 61]. This can be often supported by the previous phytochemical screening and histopathological analysis.

Hydromethanolic crude extract was fractionated with different solvents like hexane, chloroform, and water to identify which fraction of the plant extract was accountable for wound healing. The fractionation result showed that the best yield obtained from the aqueous fraction is additionally due to high concentration of polar compounds in leaves of *B. abyssinica* and was better dissolved in water. A previous study on the leaf part also demonstrated a more robust yield using aqueous solvent [40, 43]. In excision wound model, all three solvent fractions ointment preparations showed significant wound contraction as compared with negative control with different degrees. However, higher wound contraction was observed in aqueous fraction (10% w/w and 5% w/w) ointment preparation followed by chloroform fraction ointment.

The finding showed that mice treated with 10% w/w (aqueous, chloroform, and hexane) fraction ointment showed significant (*p* < 0.01) wound contraction starting from (2nd, 4th, and 12th postwounding day onwards, respectively) as compared with negative control. Wound contractions observed in 10% and 5% w/w aqueous fraction ointment treated mice were enhanced as compared with chloroform and hexane fraction ointment treated groups for several postwounding days and slightly beyond reference drug treated mice. On the other hand, the number of epithelization for excision wound model was significantly reduced from negative control (18.7) to aqueous fraction ointment 10% w/w and nitrofurazone 0.2% w/v ointment (13.5) days. Better wound healing effect of aqueous fraction could result from antibacterial, antioxidant, and antifungal activity. This will be supported by previous in vitro studies of aqueous fraction of *B. abyssinica* that had bactericidal activity against all multiresistant strains of *Staphylococcus aureus*, and aqueous fraction possesses an antifungal activity [9]. Infection can seriously delay healing process by causing poor quality animal tissue formation, reduced durability of animal tissue, and impaired epithelization [36]. Therefore antibacterial and antifungal activity of the plant is additionally important to spice up wound healing by preventing infection.

It may even ensue the antioxidant activity. This effect is supported by previous in vitro studies of aqueous fraction showed highest free radical-scavenging ability by using DPPH [43]. Antioxidants could be a defense mechanism through which an individual's body is getting protected against oxidative damage of free radicals. Free radicals are produced in response to cutaneous injury. They delay the healing process by causing damage to cellular membranes, DNA, proteins, lipids, and other cellular structure and function [52].

The antioxidant effect might enable the leaves of the experimental plant inhibit lipid peroxidation, prevent cell damage, and increase collagen fibrillary survival of the healing wound that would produce rapid wound contraction and shorter period of epithelization [62]. In addition, the higher wound healing effect of the aqueous fraction could even be related with the discharge profile from simple ointment. The efficacy and delivery of a drug are laid low with the formulation vehicle. Polar ingredient of the drug is additionally released better from the nonpolar base and contrariwise, so that most polar active ingredients within the aqueous fraction are additionally better released from nonpolar base [45].

Moderate wound contraction and moderate reduction in epithelization period of chloroform fraction could even be due to moderate antibacterial effect of the plant and fewer phytochemical content than hydromethanolic crude extracts. This is often evidenced by in vitro antibacterial studies against wound infecting bacteria. On this study, chloroform fraction had good effect in *Escherichia coli* only but moderate effect for the above explained bacteria. Previous phytochemical studies of chloroform fraction of the plant showed the presence of few phytochemicals namely flavonoids, glycosides, and alkaloid only as compared with hydromethanolic extract that contains 11 phytochemicals that are important for wound healing^9^. The late and fewer wound healing effect of hexane fraction from chloroform and water fractions could even be due to difference in the proportion (concentration) of the active components liable for anti-inflammatory activity resulting from the difference in solubility. This indicated firmer wound healing phytochemical quantitatively and/or qualitatively resides within semipolar and polar solvents.

In the above three wound healing models, higher (10% w/w) ointment preparation showed better effect than low (5% w/w) ointment preparation of the plant. This might result in increasing the dose of the plant and increase the proportion of the chemical ingredients with pharmaceutical value in plant [63].

Wound healing effect of *B. abyssinica* leaves hydromethanolic crude extract and solvent fractions could even be because of its phytoconstituents like flavonoids, saponin, tannin, triterpenoids, alkaloid, phytosterols, glycosides, phenols, steroids, anthraquinones, coumarin individual, or additive effects [64]. This is often supported by plants, which had wound healing effect in vivo and had these phytochemicals. Phytochemicals contribute to the observed wound healing effects with different mechanisms.

Flavonoids can reduce highly oxidizing free radicals by forming less reactive flavonoid radicals. As a result, they are able to prevent lipid peroxidation, increase collagen synthesis, promote the cross-linking of collagen, shorten the inflammation period, and render resistance against infections, which are important factors in enhancing the wound healing process [9, 61]. Phenolic compounds also exhibit a considerable radical-scavenging (antioxidant) activity [9]. Saponins have antioxidant, hemolytic activities precipitate proteins and aggregate red blood cells and wont to treat wounds and stop bleeding from injured site [33–35]. Saponins enhance wound contraction and bringing collagen deposition and promote angiogenesis during wound repair [45–47]. Therefore, *B. abyssinica* is employed to treat wounds in traditional medicine because of the presence of saponins. Tannins are reported as having astringent activities, which helps quicken wound healing and treat inflammations, promote wound contraction, improve healing rate, and promote healing of infectious wounds [7]. Triterpenoids are also known to stimulate the wound healing process mainly thanks to their astringent and antimicrobial property, which seems to be chargeable for wound contraction and increased rate of epithelialization. Alkaloids are observed to stimulate early phases of wound healing with the potential to stimulate fibroblasts 56. Phytosterols are credited for wound healing due to free radical-scavenging and antioxidant activity, which are known to decrease lipid peroxidation, thereby reducing cell necrosis and improving vascularity. Glycosides possess antioxidant, antimicrobial, and anti-inflammatory effects [47, 65]. Steroids are antimicrobial, analgesic, anti-inflammatory, and anthraquinones modulate inflammation by partially inhibiting cyclooxygenase [66].

## 8. Conclusion

The hydromethanolic crude extract and solvent fractions of *B. abyssinica* leaves showed wound healing effects on tested parameters in mice. Aqueous fractions possess higher wound healing activity than chloroform and hexane fractions. This indicated that phytochemicals present in the leaves were soluble in the solvents used for extraction furthermore as for fractionation. This upholds its folkloric use of leaves of *B. abyssinica* and might be considered as a possible source to develop new wound healing agent.

## Figures and Tables

**Table 1:** Simple ointment formula [42].

Ingredients	Master formula (MF) (g)	Reduced formula (RF) (g)
Wool fat	50	15
Hard paraffin	50	15
Cetostearyl alcohol	50	15
White petrolatum	850	255
Total	1000	300

**Table 2:** Yield of 80% hydromethanolic leaf crude extract and solvent fractions of *B. abyssinica* leaf.

Name of extract	Amount (g)	Yield in grams	Percent yield
Crude extract	1000	173	17.3
Aqueous fraction	70 grams was dissolved in water	45	64.3
Chloroform fraction		7.5	10.7
Hexane fraction		2.5	3.6

**Table 3:** Effect of topical application of ointment prepared from 80% hydromethanolic leaf crude extract of *B. abyssinica* on wound area (mm^2^) in excision wound model.

Ointment	Day 4	Day 6	Day 8	Day 10	Day 12	Day 14	Day 16
SOB	236.00 ± 4.62	175.67 ± 4.77	132.17 ± 2.97	109.50 ± 2.86	76.33 ± 1.89	44.83 ± 1.82	24.00 ± 1.67
HCEO 5% w/w	218.17 ± 3.48^a1^	158.67 ± 3.86^a2^	115.67 ± 3.61^a2c3d3^	57.33 ± 2.92^a3c3d3^	24.50 ± 0.99^a3c3d3^	7.67 ± 0.49^a3c2d2^	1.17 ± 0.54^a3^
HCEO 10% w/w	209.33 ± 4.12^a2^	146.00 ± 3.08^a3^	89.50 ± 1.84^a3b3^	39.17 ± 0.95^a3b3^	14.83 ± 0.98^a3b3^	1.67 ± 0.56^a3b2^	0.00 ± 0.00^a3^
NO 0.2% w/v	210.83 ± 3.64^a2^	144.83 ± 3.45^a3^	92.67 ± 2.04^a3b3^	40.67 ± 1.61^a3b3^	16.00 ± 0.73^a3b3^	1.83 ± 0.65^a3b2^	0.00 ± 0.00^a3^

Data are expressed as mean ± SEM; *n* = 6, a, compared with simple ointment; b, compared with crude methanolic extract 5% (w/w); c, compared with crude methanolic extract 10% (w/w) ointment; d, compared with nitrofurazone 0.2%(w/v) ointment. 1, *p* < 0.05, 2, *p* < 0.01, and 3, *p* < 0.001. SOB, simple ointment base; HCEO, hydromethanolic crude extract of *B. abyssinica* leaves; NO, nitrofurazone ointment.

**Table 4:** Effect of topical application of ointment prepared from 80% hydromethanolic leaves crude extract of *B. abyssinica* on percent wound contraction in excision wound model.

Ointment	Day 4	Day 6	Day 8	Day 10	Day 12	Day 14	Day 16
SOB	7.60 ± 1.06	31.27 ± 1.06	48.27 ± 0.54	57.11 ± 0.98	70.1 ± 0.67	82.45 ± 0.62	90.63 ± 0.54
HCEO 5% w/w	16.12 ± 0.78^a3^	39.00 ± 1.22^a3d1^	55.56 ± 1.04^a3c3d3^	77.99 ± 0.92^a3c3d3^	90.58 ± 0.35^a3c3d3^	97.05 ± 0.20^a3c2d2^	99.55 ± 0.21^a3^
HCEO 10% w/w	17.69 ± 0.84^a3^	42.60 ± 0.45^a3b3^	64.81 ± 0.41^a3b3^	84.6 ± 0.25^a3b3^	94.18 ± 0.32^a3b3^	99.35 ± 0.22^a3b2^	100 ± 0.00^a3^
NO 0.2% w/v	17.46 ± 0.34^a3^	43.31 ± 0.79^a3b3^	63.7 ± 0.75^a3b3^	84.09 ± 0.53^a3b3^	93.73 ± 0.28^a3b3^	99.29 ± 0.26^a3b2^	100 ± 0.00^a3^

Data are expressed as mean ± SEM; *n* = 6; a, compared with simple ointment; b, compared with crude methanolic extract 5%(w/w); c, compared with crude methanolic extract 10% (w/w) ointment; d, compared with nitrofurazone 0.2%(w/v) ointment. 1, *p* < 0.05; 2, *p* < 0.01; 3, *p* < 0.001. SOB, simple ointment base; HCEO, hydromethanolic crude extract of *B. abyssinica* leaves; NO, nitrofurazone ointment.

**Table 5:** Effect of topical application of ointment prepared from 80% hydromethanolic leaf crude extract of *B. abyssinica* on epithelization period in excision wound model.

Treatment groups	Epithelization period (days)	Percentage reduction epithelization periods
Simple ointment base	19.5 ± 1.64	—
Hydromethanolic crude extract ointment 5% w/w	15.83 ± 0.40^a3^	18.8
Hydromethanolic crude extract ointment 10% w/w	14 ± 0.37^a3^	28.2
Nitrofurazone ointment 0.2% (w/v)	14.17 ± 0.40^a3^	27.4

Data are expressed as mean ± SEM; *n* = 6; SOB = simple ointment base, HCEO = hydromethanolic crude extract ointment, NO = nitrofurazone ointment; a, compared with simple ointment; b, compared with crude methanolic extract 5% (w/w); c, compared with crude methanolic extract 10% (w/w) ointment; d, compared with nitrofurazone 0.2% (w/v) ointment. 1, *p* < 0.05; 2, *p* < 0.01; 3, *p* < 0.001.

**Table 6:** Histological qualitative determination of wound healing processes and healing phases of *B. abyssinica* crude extract ointment in excision wound model in mice.

Group	Inflammatory cells	Fibroblast proliferation	Collagen depositions	Mononuclear cells	Polymorphonuclear cells	Neovascularization
SOB	+++	+	+	—	+++	—
HCEO 5%	+	++	++	—	—	+
HCEO 10%	—	+++	+++	—	—	—
NO 0.2%	—	+	+	—	+	+

Low concentration (+), moderate concentration (++), and high concentration (+++) for epidermal and/or dermal remodeling. SOB, Simple ointment base; HCEO, hydromethanolic crude extract of *B. abyssinica* leaves; NO, nitrofurazone ointment.

**Table 7:** Effect of topical application of hydromethanolic crude extract of *B. abyssinica* leaves on tensile strength in incision wound model in mice.

Treatment groups	Tensile strength in grams	Percent tensile strength
Untreated	210.5 ± 3.03	—
Simple ointment base	240.33 ± 6.42	14.17
Hydromethanolic crude extract ointment 5%w/w	354.5 ± 9.2^a3b3^	47.5
Hydromethanolic crude extract ointment 10%w/w	387.33 ± 16.61^a3b3^	61.17
Nitrofurazone ointment 0.2% w/v	390.83 ± 10.39^a3b3^	62.62

Data are expressed as mean ± SEM; *n* = 6; **a**, compared with untreated group; **b**, compared with simple ointment; 3, *p* < 0.001.

**Table 8:** Effect of topical application of ointment prepared from 80% hydromethanolic crude extract of *B. abyssinica* leaves on wound area contraction (mm^2^) in burn wound model in mice.

Days	Simple ointment	Methanolic crude extract 5% (w/w)	Methanolic crude extract 10% (w/w)	Nitrofurazone 0.2% (w/v)
2	282.33 ± 4.89	272.17 ± 5.24	272.00 ± 5.05	271.83 ± 4.73
4	254.33 ± 5.10	237.17 ± 4.21	233.17 ± 4.48^a1^	232.00 ± 4.12^a1^
6	227.00 ± 3.69	208.00 ± 3.25^a1c1d2^	190.67 ± 4.56^a3b2^	186.83 ± 4.44^a3b2^
8	195.50 ± 4.46	167.67 ± 3.14^a1c1d2^	150.67 ± 3.25^a3b1^	144.00 ± 4.07^a3b2^
10	165.83 ± 3.83	134.83 ± 2.77^a3c2d3^	117.83 ± 1.54^a3b2^	113.50 ± 2.77^a3b3^
12	135.00 ± 3.77	99.00 ± 3.07^a3c1d2^	86.33 ± 3.20^a3b2^	83.50 ± 1.93^a3b2^
14	105.17 ± 3.79	62.50 ± 3.11^a3c2d2^	50.17 ± 2.40^a3b2^	48.00 ± 1.83^a3b2^
16	76.67 ± 2.49	33.67 ± 2.99^a3d2^	26.00 ± 1.39^a3^	21.83 ± 1.56^a3b2^
18	49.50 ± 3.63	9.83 ± 1.47^a3d2^	3.33 ± 0.76^a3^	1.33 ± 0.71^a3b2^
20	17.67 ± 3.03	0.50 ± 0.34^a3^	0.00 ± 0.00^a3^	0.00 ± 0.00^a3^
22	3.50 ± 1.23	0.00 ± 0.00^a2^	0.00 ± 0.00^a2^	0.00 ± 0.00^a2^
24	0.00 ± 0.00	0.50 ± 0.34	0.00 ± 0.00	0.00 ± 0.00

Data are expressed as mean ± SEM; *n* = 6, a, compared with simple ointment; b, compared with crude methanolic extract 5% (w/w); c, compared with crude methanolic extract 10% (w/w) ointment; d, compared with nitrofurazone 0.2% (w/v) ointment. 1, *p* < 0.05; 2, *p* < 0.01; 3, *p* < 0.001.

**Table 9:** Effect of topical application of ointment prepared from 80% hydromethanolic leaf crude extract of *B. abyssinica* on percent wound contraction in burn wound model.

Data collection days	Simple ointment	Hydromethanolic crude extract 5% (w/w)	Hydromethanolic crude extract 10% (w/w)	Nitrofurazone ointment 0.2% (w/v)
2	0.00	0.00	0.00	0.00
4	9.93 ± 0.54	12.84 ± 0.58^a1^	14.25 ± 1.02^a1^	14.65 ± 0.64^a1^
6	19.59 ± 0.25	23.52 ± 1.00^a1c2d3^	29.87 ± 1.42^a3b2^	31.29 ± 0.82^a3b3^
8	30.75 ± 1.04	38.35 ± 1.08^a2c2d3^	44.52 ± 1.52^a3b2^	47.04 ± 1.00^a3b3^
10	41.26 ± 0.93	50.41 ± 1.10^a3c2d3^	56.57 ± 1.25^a3b2^	58.26 ± 0.56^a3b3^
12	52.15 ± 1.27	63.6 ± 1.05^a3c1d2^	68.17 ± 1.47^a3b1^	69.27 ± 0.56^a3b2^
14	62.72 ± 1.33	77.08 ± 0.85^a3c1d2^	81.47 ± 1.14^a3b1b1^	82.34 ± 0.59^a3b2^
16	72.82 ± 0.88	87.71 ± 0.89^a3d2^	90.42 ± 0.57^a3^	91.97 ± 0.53^a3b2^
18	82.45 ± 1.28	96.43 ± 0.48^a3d1^	98.76 ± 0.29^a3^	99.52 ± 0.26^a3b1^
20	93.7 ± 1.10	99.82 ± 0.12^a3^	100 ± 0.00^a3^	100 ± 0.00^a3^
22	98.75 ± 0.43	100 ± 0.00^a2^	100 ± 0.00^a2^	100 ± 0.00^a3^
24	100 ± 0.00	99.82 ± 0.12	100 ± 0.00	100 ± 0.00

Data are expressed as mean ± SEM; *n* = 6; a, compared with simple ointment; b, compared with crude methanolic extract 5% (w/w); c, compared with crude methanolic extract 10% (w/w) ointment; d, compared with nitrofurazone 0.2% (w/v) ointment. 1, *p* < 0.05; 2, *p* < 0.01; 3, *p* < 0.001.

**Table 10:** Histological qualitative determination of wound healing processes and healing phases of *B. abyssinica* leaf hydromethanolic crude extract ointment in partial thickness wound model in mice.

Group	Inflammatory cells	Fibroblast proliferation	Collagen depositions	Mononuclear cells	Polymorphonuclear cells	Neovascularization
SOBS	+++	+	+	—	+++	+
HCEO 5%	+	+	+	+	+	+
HCEO10%	+	+	++	+	+	++
NO 0.2%	+	++	++	+	+	++

Low concentration (+), moderate concentration (++), and high concentration (+++) for epidermal and/or dermal remodeling. SOB, Simple ointment base; HCEO, hydromethanolic crude extract of *B. abyssinica* leaves; NO, nitrofurazone ointment.

**Table 11:** Effect of topical application of ointment prepared from solvent fractions of *B. abyssinica* leaves on wound area contraction (mm^2^) in excision wound model in mice.

Ointment	Day 4	Day 6	Day 8	Day 10	Day 12	Day 14	Day 16
SOB	258.33 ± 2.67	224.33 ± 2.55	184.17 ± 4.22	148.00 ± 2.88	106.17 ± 2.68	68.67 ± 2.77	32.50 ± 1.29
AQF 5% w/w	233.17 ± 1.96^a3^	195.50 ± 4.24^a3^	148.17 ± 2.17^a3^	88.50 ± 1.96^a3^	39.33 ± 0.67^a3^	2.17 ± 1.11^a3^	0.00 ± 0.00^a3^
AQF 10% w/w	227.83 ± 1.92^a3^	195.00 ± 3.83^a3^	146.17 ± 1.30^a3^	86.83 ± 1.70^a3^	38.67 ± 1.05^a3^	1.67 ± 0.76^a3^	0.00 ± 0.00^a3^
CHF 5% w/w	243.67 ± 3.40^a1d2^	215.67 ± 1.84^a1d3^	169.83 ± 2.01^a1d3^	126.17 ± 1.54^a3d3^	75.17 ± 1.54^a3d3^	27 ± 2.16^a3d3^	2.33 ± 0.84^a3^
CHF 10% w/w	241.83 ± 3.79^a2d1^	212.50 ± 1.23^a2d3^	164.33 ± 3.16^a2d2^	124.00 ± 2.75^a3d3^	73 ± 1.88^a3d3^	24.83 ± 1.62^a3d3^	1.83 ± 0.91^a3^
HF 5% w/w	246.33 ± 4.86^d2^	218.33 ± 5.06^d3^	183.33 ± 4.02^d3^	135.67 ± 2.06^a2d3^	82.33 ± 1.80^a3d3^	39.5 ± 1.29^a3d3^	5.00 ± 0.58^a3d2^
HF 10% w/w	248.00 ± 3.92^d2^	217.83 ± 2.70^d2^	181.50 ± 4.05^d3^	133.00 ± 1.59^a3d3^	79.5 ± 1.48^a3d3^	36.67 ± 1.05^a3d3^	4.67 ± 0.56^a3d2^
NO 0.2% w/v	228.00 ± 2.08^a3^	195.33 ± 2.28^a3^	147.67 ± 2.23^a3^	87.33 ± 1.17^a3^	40.5 ± 1.61^a3^	2 ± 0.93^a3^	0.00 ± 0.00^a3^

Data are expressed as mean ± SEM; *n* = 6; a, compared with simple ointment; b, compared with fraction 5% (w/w); c, compared with fraction 10% (w/w) ointment; d, compared with nitrofurazone 0.2% (w/v) ointment. 1, *p* < 0.05; 2, *p* < 0.01; 3, *p* < 0.001. AQF, aqueous fraction of hydromethanolic crude extract of *B. abyssinica* leaves; CHF, chloroform fraction of hydromethanolic crude extract of *B. abyssinica* leaves; HF, hexane fraction of hydromethanolic crude extract of *B. abyssinica* leaves; NO, nitrofurazone ointment.

**Table 12:** Effect of topical application of ointment prepared from solvent fractions of *B. abyssinica* leaves on percent wound contraction in burn wound model in mice.

Ointment	Day 4	Day 6	Day 8	Day 10	Day 12	Day 14	Day 16
SOB	7.58 ± 0.28	19.74 ± 0.45	34.12 ± 1.24	47.05 ± 0.93	62.02 ± 0.83	75.45 ± 0.91	88.37 ± 0.44
AQF 5% w/w	12.16 ± 1.03^a1^	26.3 ± 2.08^a2^	44.16 ± 1.16^a3d1f3g3^	66.66 ± 0.75^a3d3e3f3g3^	85.18 ± 0.35^a3d3e3f3g3^	99.17 ± 0.43^a3d3e3f3g3^	100 ± 0.00^a3f3g3^
AQF 10% w/w	13.72 ± 0.74^a1^	26.17 ± 1.26^a2^	44.64 ± 0.57^a3d2f3g3^	67.12 ± 0.60^a3b3c3h3^	85.35 ± 0.43^a3d3e3f3g3^	99.38 ± 0.28^a3d3e3f3g3^	100 ± 0.00^a3f3g3^
CHF 5% w/w	11.32 ± 0.91	21.49 ± 0.64	38.15 ± 1.16^b1c2h1^	54.05 ± 0.86^a3b3c3h3^	72.65 ± 0.38^a3b3c3h3^	90.17 ± 0.78^a3b3c3f2g2h3^	99.16 ± 0.30^a3^
CHF 10% w/w	12.21 ± 0.99	22.81 ± 1.04	40.26 ± 1.72^a1f1g1^	54.93 ± 1.30^a3b3c3e1h3^	73.46 ± 0.88^a3b3c3f1h3^	91.01 ± 0.50^a3b3c3f3g3h3^	99.32 ± 0.33^a3^
HF 5% w/w	11.53 ± 1.11	21.6 ± 1.26	34.15 ± 1.16^b3c3e1h3^	51.24 ± 0.81^a1b3c3e1h3^	70.41 ± 0.66^a3b3c3e1h3^	85.81 ± 0.44^a3b3c3d3e3h3^	98.21 ± 0.20^a3b3c3e1h3^
HF 10% w/w	10.59 ± 0.86	21.46 ± 0.13	34.58 ± 1.00^b3c3e1h3^	52.04 ± 0.30^a2b3c3h3^	71.32 ± 0.62^a3b3c3h3^	86.78 ± 0.36^a3b3c3d2e3h3^	98.32 ± 0.20^a3b3c3h3^
NO 0.2% w/v	14.09 ± 1.19	26.41 ± 1.00^a2^	44.36 ± 0.95^a3d1f2g2^	67.1 ± 0.47^a3d3e3f3g3^	84.75 ± 0.59^a3d3e3f3g3^	99.25 ± 0.35^a3d3e3f3g3^	100 ± 0.00^a3f3g3^

Data are expressed as mean ± SEM; *n* = 6; a, compared with simple ointment; b, compared with aqueous fraction 5% (w/w); c, compared with aqueous fraction 10% (w/w) ointment; d, compared with chloroform fraction 5% (w/w); e, compared with chloroform fraction 10% (w/w) ointment; f, compared with hexane fraction 5% (w/w) ointment; g, compared with hexane fraction 10% (w/w) ointment; h, compared with nitrofurazone 0.2% (w/v) ointment. 1*p* < 0.05, 2*p* < 0.01, 3*p* < 0.001. AQF, aqueous fraction of hydromethanolic crude extract of *B. abyssinica* leaves; CHF, chloroform fraction of hydromethanolic crude extract of *B. abyssinica* leaves; HF, hexane fraction of hydromethanolic crude extract of *B. abyssinica* leaves; NO, nitrofurazone ointment.

**Table 13:** Effect of topical application of ointment prepared from solvent fractions of 80% hydromethanolic leaf crude extract of *B. abyssinica* on period of epithelization on excision wound model in mice.

Treatment	Epithelization period in days	Percentage reduction in period epithelization
Simple ointment	18.67 ± 0.33	0.00
Aqueous fraction 5% (w/w) ointment	13.67 ± 0.21^adefg(3)^	26.78
Aqueous fraction 10% (w/w) ointment	13.50 ± 0.34^adefg(3)^	27.69
Chloroform fraction 5% (w/w) ointment	15.83 ± 0.40^abch(3)fg(1)^	15.21
Chloroform fraction 10% (w/w) ointment	15.67 ± 0.33^abch(3)f2g1^	16.07
Hexane fraction 5% (w/w) ointment	17.33 ± 0.21^ad(1)bch(3)e2^	7.18
Hexane fraction 10% (w/w) ointment	17.17 ± 0.17^ade(1)bch(3)^	8.03
Nitrofurazone 0.2% (w/v) ointment	13.50 ± 0.22^adefg(3)^	27.69

Data are expressed as mean ± SEM; *n* = 6; a, compared with simple ointment; b, compared with 5% aqueous fraction; c, compared with 10% aqueous fraction; d, compared with 5% chloroform fraction; e, compared with 10% chloroform fraction; f, compared with 5% hexane fraction; g, compared with 10% hexane fraction; h, compared with nitrofurazone 0.2% ointment; 1, *p* < 0.05; 2, *p* < 0.01; 3, *p* < 0.001.

## Data Availability

The datasets used and/or analyzed during the current study are available from the corresponding author and will be submitted when requested.
